# High-purity foam-like micron-sized gold cage material with tunable plasmon properties

**DOI:** 10.1038/s41598-020-72831-9

**Published:** 2020-10-06

**Authors:** Shuo Dong, Lin Yi, Lexiao Cheng, Shijian Li, Weiming Yang, Zhebin Wang, Shaoen Jiang

**Affiliations:** 1 School of Physics, Huazhong University of Science and Technology, Wuhan, 430074 Hubei China; 2 Laser Fusion Research Center, China Academy of Engineering Physics, Mianyang, 621900 Sichuan China

**Keywords:** Surfaces, interfaces and thin films, Composites

## Abstract

Herein, by growing mono dispersed gold nanoparticles (MNPAu) on the surface of polystyrene (PS)/nanogold (Au) core–shell composites (PS@Au), we successfully synthesized a micron-sized gold cage (2.6–10.7 μm), referred to as PS@Au@MNPAu for the first time. The new micron-gold cage materials exhibit broadband absorption range from near-ultraviolet to near-infrared, which is unlike the conventional nanogold core–shell structure. The uniform growth of MNPAu on the surface forms a new photonic crystal spectrum. The strong coupling of the spectra causes anomalous absorption in the ultraviolet-near infrared band (400–900 nm). Furthermore, by removing the PS core, a nanogold cavity structure referred to as Au@MNPAu was prepared. This structure demonstrated a high purity (> 97 wt%), low density (9–223 mg/cm^3^), and high specific surface area (854 m^2^/g). As the purification process progressed, the MNPAu coupling on the surface of the micro-gold cage strengthened, resulting in the formation of peaks around 370 nm, plasma resonant peaks around 495 nm, and structural bands of photonic crystal peaks around 850 nm. The micron-sized gold cage provides hybridized and tunable plasmonic systems. The theoretical simulations indicate that this plasmon anomalous absorption phenomena can be understood as the novel form of the topological structural transitions near the percolation threshold, which is consistent experimental measurements.

## Introduction

A core–shell structure refers to a crystalline, amorphous, or polycrystalline structure that contains a spherical particle of submicron size as the core that is coated with nanoparticles using physical and chemical methods^1^. The design and synthesis of monodispersed, flexible, and controllable core–shell structural materials are some of the main focuses of research in the field of modern material physics^2^. Composite structural materials have wide application potential in photoelectricity^3^, catalysis^4^, biomedicine^5^, environmental science^6^, energy^7^, and other fields.

Following the pioneering work of Halas et al. in employing a seed-mediated growth approach to synthesize nanogold^8^ and nano-silver shells^9^, numerous studies have been conducted to improve the synthesis process^10^, explore the structure–property relationship^11^, and extend the potential applications of these particles^12,13^. Due to the excellent optical properties of metallic nanoshells, electrodynamic simulations have been extensively compared with experimental results to characterize shell thickness and other geometric parameters^14^. In addition, by assembling noble metal nanocomposites onto the surface of a material with a specific property, it is possible to obtain a dual-functional composite material^15^. One of the most common methods for constructing gold nanoshells is to attach small gold particles to the surface of a silica core and then use these sites as seeds to induce shell growth^8,16^. Ideally, this approach should be performed based on a theoretical understanding of the spectral behavior of the particles. This process has been proven feasible by the analytical solution to the problem, which includes the scattering of electromagnetic waves from a coated sphere^17^, as elaborated in several studies^18,19^. In short, the plasmon band position of the particles is blue-shifted with the increase in shell thickness. However, this is observed only when the shell thickness is smaller than the radius of the underlying sphere^8^.

Studies have shown that core–shell materials are generally nanosized. For example, Shi et al.^20^ elaborated on the characteristic plasmon resonance absorption peaks of PS@Au without studying the materials at the microscale. Therefore, it is necessary to further study the optical properties of noble metal core–shell structures. In this study, PS@Au microspheres with different polycrystalline structures were synthesized through seed-mediated growth using the Frens method by adjusting the concentration of tetrachloroauric acid and the reducing agent.

The preparation of composite gold foam can be achieved by assembling nanogold particles. For example, Mezzenga et al.^21^ used amyloid nanofibers as a scaffold to load AuNPs, leading to the formation of gold crystal amyloid composite aerogels with densities as low as 5.8 mg/cm^3^. Further, the preparation of high-purity foam gold materials can be achieved by template-assisted electrochemical deposition. This was demonstrated by Nyce et al. who used closely-packed polystyrene beads as a sacrificial template for electroplating gold^22,23^, achieving gold foams a density of 280 mg/cm^3^ and tunable pore sizes. Despite these achievements, no studies have demonstrated gold aerogels that simultaneously exhibit suitable properties such as ultra-low densities, high purity, and high specific surface areas.

**Figure 1:**
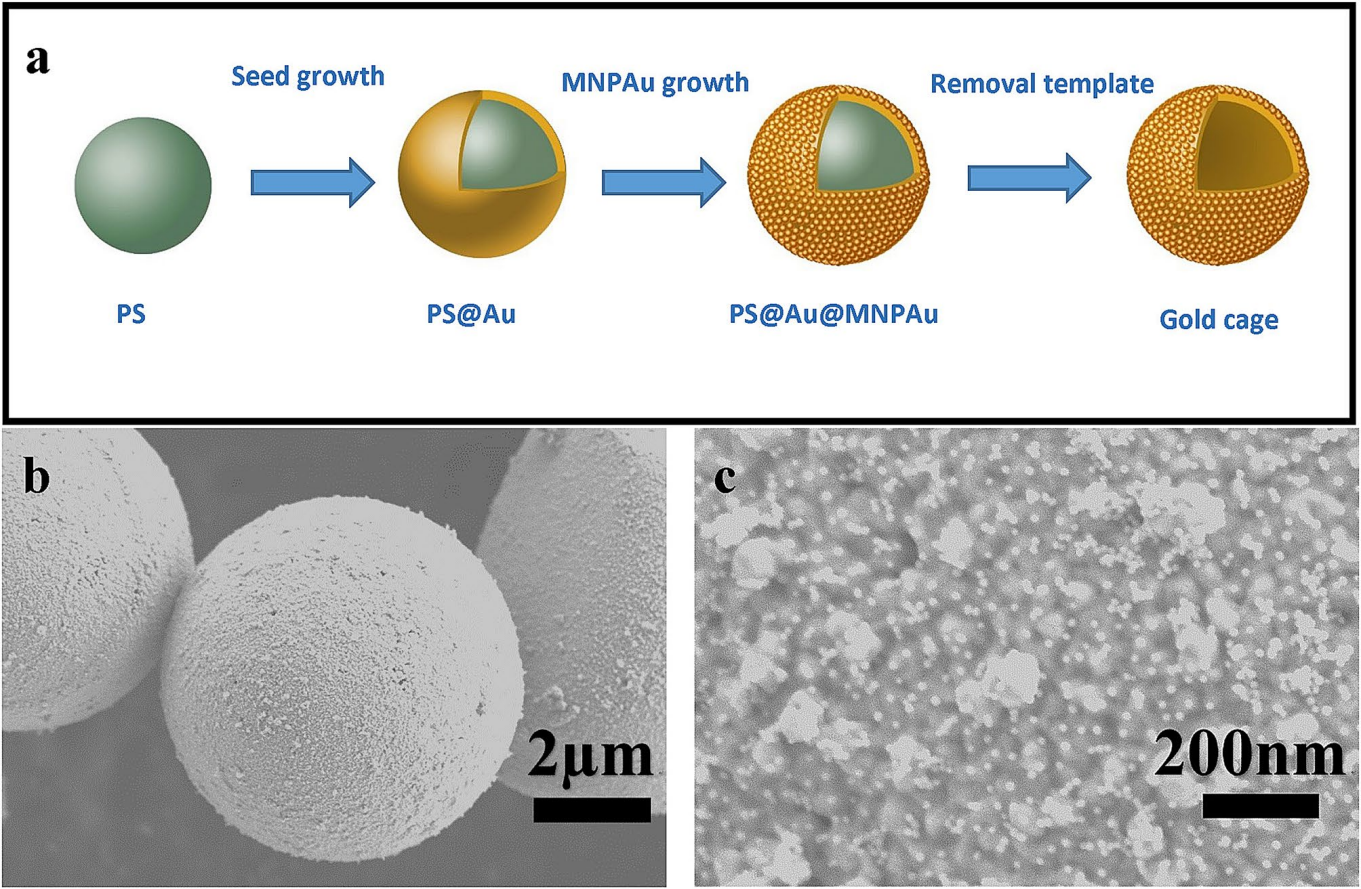
(**a**) Synthesis of a micron-sized gold cage (Adobe Photoshop 19.0 https://www.photoshop.com/en), (**b**,**c**) SEM characterization of a micron-sized gold cage.

In this study (Fig. 1a), by improving the surface growth process of the Frens method, polystyrene (PS)/nanogold (Au) core–shell composites (PS@Au) with particle sizes in the micron order were synthesized, and the changes of the LSPR peak position, in the cases of complete and incomplete shell structures were investigated. Next, mono-dispersed nanoparticles of gold (MNPAu) were grown on the outer surface of PS@Au to ensure uniformity in particle size during seed-mediated growth (Fig. 1b,c). That is, through the joint application of the seed-mediated and particle-mediated growth methods, a micron-sized gold cage with uniformly distributed nanogold on the surface, referred to as PS@Au@MNPAu, was successfully synthesized. Compared with PS@Au synthesized through surface growth in the Frens method, the PS@Au@MNPAu exhibited the notable property of broadband absorption. It is observable that there exist the monodispersed and the non-monodispersed growths to form the golden nanoparticles and the clusters in the crossovers on the surface, as shown in Fig. 1c. Further, various types of impurities generated during the synthesis of PS@Au@MNPAu were removed using an impurity-specific purification method, resulting in a micron-sized gold cage with high purity, low density, and high specific surface area. With the removal of the PS core, this novel nanogold-containing cavity structure exhibited enhanced broadband absorption.

In conventional approximations, such as the Mie-Maxwell–Garnett theories^24–26^ and the Gorkov-Eliashberg (GE) quantum theory for gold particles in the Gaussian symplectic ensemble^27–29^, an effective dielectric function has been derived considering the variations in sample geometries and sizes, such as the fractal and the percolation, the dielectric and the impurity environments within the incident light field^8,12^. This has been achieved by considering the effects of the depolarizing field of the monodispersed gold particles and the non-mono-dispersed gold clusters (Fig. 1b,c), the Fano resonances, and multiscale Gaussian distributions such as nanoparticles and core–shell structures^18–20^. Corresponding to the various aforementioned experimental stages, numerical simulations have been carried out for examining the absorbance of the gold cages to describe hybridized and tunable absorption in the ultraviolet-near infrared range at structural transitions. This is consistent with the experimental observations and measurements of plasma resonances and photonic crystal band crossovers.

## Results and discussions

**Figure 2:**
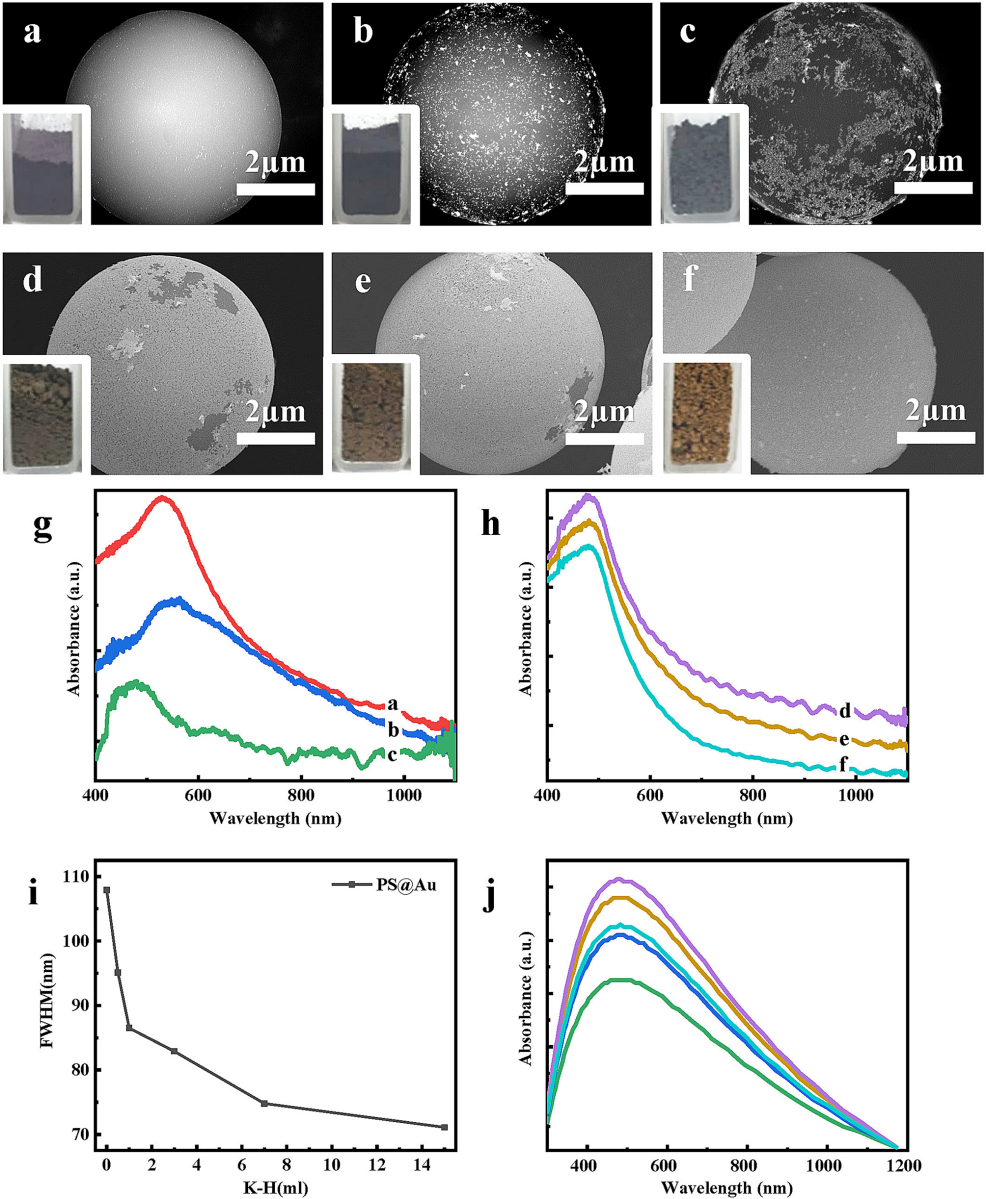
SEM images (**a**–**f**) and UV–Vis characterization (**g**,**h**) of PS@Au synthesized with 0 (red), 0.5 (blue), 1 (green), 3 (purple), 7 (yellow), and 15 ml (cyan) of K–H solution, respectively. (**i**) The FWHM of the absorption peak of the UV spectrum in PS@Au material varies with the K–H solution. (**j**) Simulation of absorbance as a function of the filling factor of clusters ƒ_c_ = 0.77, 0.79, 0.81, 0.83, 0.85 and the ratio R_i_/R_0_ = 0.81, 0.85, 0.89, 0.92, 0.96 at d = 5 nm and Λ = 1.

First, we describe the growth of the PS@Au according to the steps in Fig. 1a. For the incomplete shell structures (Fig. 2a–c), in fractal geometries, the nanogold particles formed island-like agglomerates with an increase in the volume of the K–H (K_2_CO_3_ and HAuCl_4_ mixed solution) solution. Because of the non-uniform particle size distribution of nanogold particles, thereby an increased full-width at half maximum (FWHM) of the UV–Vis absorption peaks. The plasmon resonance absorption peaked at 529, 564, and 481 nm, respectively, and the peak positions were first red-shifted and then blue-shifted. The red shift was attributed to the increase in the refractive index of PS@Au, which was observed with an increase in the surface nanogold particles. Furthermore, it was ascribed to the increase in the interparticle coupling, which was observed with a decrease in the distance between the nanogold particles. This explanation was qualitatively consistent with the Mie theory and the theoretical simulation results of Pissuwan et al. in the range of 700–1200 nm for nano core-shells^30^. However, there exist considerable discrepancies in the high frequency range (< 700 nm). In the case where 1 ml K–H solution was used, the plasmon resonance absorption peak of the samples started to undergo a blue shift (from 564 to 481 nm). This implied that the samples started to undergo structural phase transitions, that is, the defects led to the formation of LSP modes. PS@Au with a complete shell (Fig. 2d–f), had a smaller FWHM than that of PS@Au with an incomplete shell (Fig. 2i), and the absorption peaks showed minor variations (approximately 481 nm) with the increase in shell thickness. This indicated a weak dependence of the LSPR peak position in the complete shells.

Thomas et al.^12^ studied the growth and optical properties of incomplete gold layers on silica particles and found that the plasmon modes of the system exhibited two bands, one in the range of 500–600 nm (“high energy”) and the other in the range of 600–800 nm (“low energy”). They also found that as the gold nanoparticles grew, the lower energy band became stronger relative to the higher energy band. Further, this was accompanied by a red shift of the bands. Pan et al.^31^ theoretically calculated the gain-assisted double plasmonic resonances to enhance second harmonic generation (SHG) in centrosymmetric multilayered nanocomposites. Moreover, PS@Au synthesized in this study showed ultra-wideband absorption due to coupling between two different layers of the nanogold shell. By considering the depolarizing effects of monodispersed gold clusters and the Fano resonances for the shell with the ratio R_i_/R_0_ > 0.81 and thickness of approximately 200 nm in the percolation regime (ƒ_c_ > 0.5), the present simulations (Fig. 2j) are consistent with the experimental measurements (Fig. 2g,h) in the high-frequency absorbance (< 400 nm), where ε_s_ = 2.5 is used in our calculations as the PS impurity is major.

**Figure 3:**
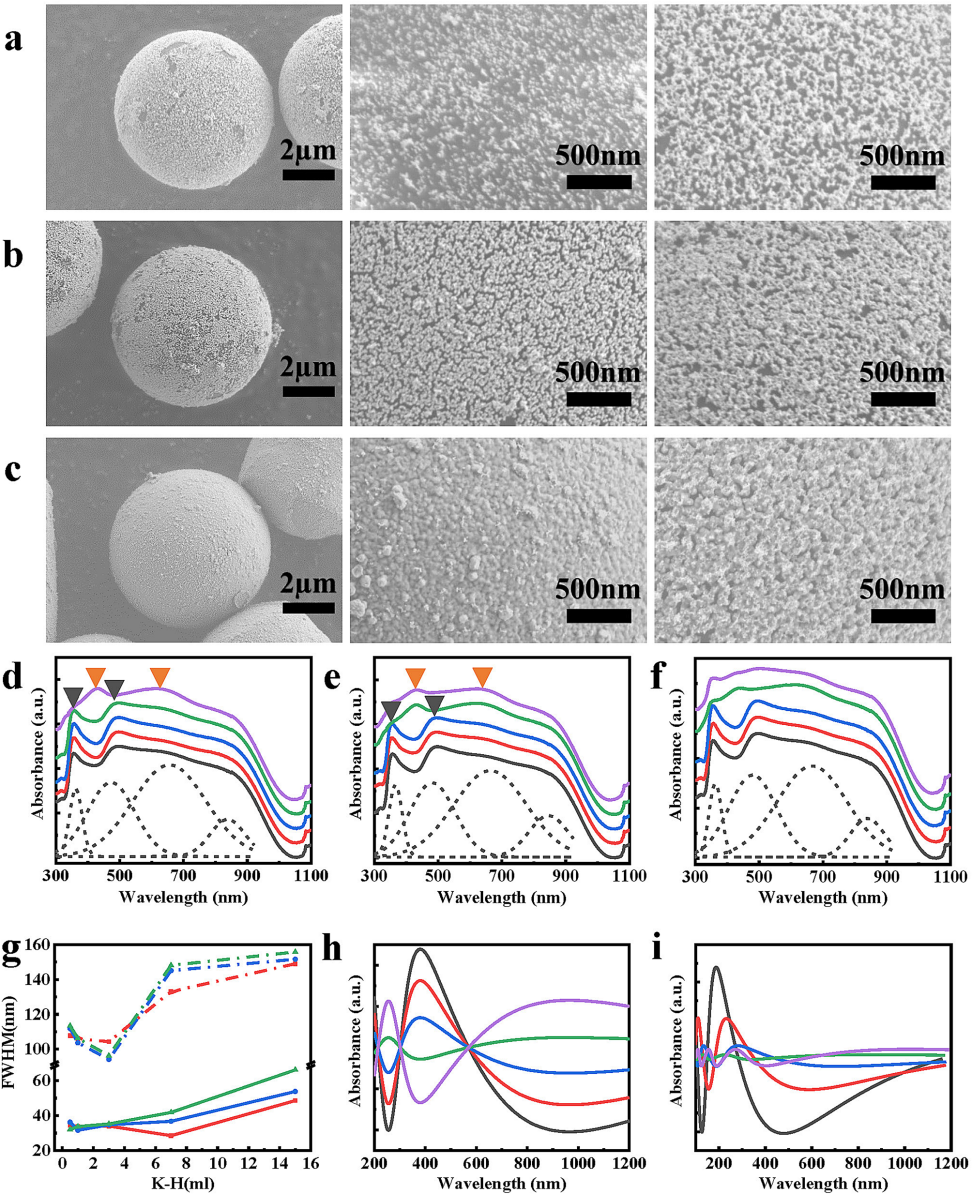
(**a**–**c**) SEM images of PS@Au@MNPAu synthesized with 0.5, 3.0, and 15.0 ml of K–H solution, and SEM images of PS@Au@MNPAu after one round and three rounds of growth of MNPAu. (**d**–**f**) UV–Vis absorption spectra of micron-sized PS@Au@MNPAu after one round, two rounds, and three rounds of growth of MNPAu. The amount of K–H solution used in the preparation of the micron gold cage is 0.5 (gray), 1.0 (red), 3.0 (blue), 7.0 (green), and 15.0 ml (purple). The dotted line in the figure is the result of the deconvolution of the solid gray line. The peaks are around 365, 479, 659, and 842 nm. (**g**) The FWHM of two absorption peaks. The growth times of MNPAu are once (red), twice (green), and three times (blue). (**h**) Absorbance as a function of ƒ_c_ = 0.38, 0.40, 0.42, 0.44, 0.46 and R_i/_R_0_ = 0.47, 0.54, 0.60, 0.66, and 0.73 at the fixed d = 5 nm and Λ = 0.80. (**i**) Absorbance as a function of d = 3.9, 4.2, 4.5, 4.8, 5.1. The others values are the same as in (**h**).

As shown in Fig. 3a, as the number of rounds of MNPAu growth increased, the number of MNPAu on the surface of PS@Au microspheres increased remarkably. Additionally, MNPAu prepared by the particle-mediated growth method were uniformly distributed on the surface of the PS@Au microspheres. This was in contrast to the seed-mediated growth method. Moreover, MNPAu showed a denser distribution on the surface of an incomplete shell structure than that of a complete shell structure. This was attributed to the electromagnetic shielding effect of the complete nanogold shell, which weakened the binding force between MNPAu and PS@Au microspheres. However, the number of MNPAu on the microsphere surface still gradually increased with the number of rounds of growth. As shown in Fig. 3a–c, the amount of MNPAu attached to the microsphere surface decreased with the increase in the thickness of the nanogold shell. It is obvious that the monodispersed and the non-monodispersed growths coexist (Fig. 3a–c) at the shell surfaces, approximated to the fractal and the percolation structural crossovers.

UV–Vis absorption of PS@Au was solely due to plasmon resonance absorption. As shown in Fig. 3d–f, compared with the PS@Au synthesized through seed-mediated growth, and the micron-sized PS@Au@MNPAu exhibited bimodal absorption when a small amount of MNPAu were grown. An optical property similar to that observed by Thomas et al.^12^ during the process of growing gold layers on silica (> 500 nm). However, plasmon anomalous absorbance exists near the ultraviolet range of approximately 370 nm. By considering the TE/TM mode hybridization and the two-point correlations, the simulations in Fig. 3h–i display the absorbance as a function of the ratio R_i_/R_0_ for the monodispersed and non-monodispersed growths below the percolation threshold at ƒ_c_ = 0.5. The absorptions exhibit the complicated orbital hybridization and the coherence superposition of the phase. This is in accordance with the experimental observations and the measurements qualitatively.

In the actual shell growth processes, with further increase in MNPAu, the UV–Vis absorption of PS@Au@MNPAu exhibited broadband absorption features (400–900 nm), which was attributed to the combination of surface plasmon excitation and MNPAu-induced excitation of photonic crystals. The presence of a nanogold shell on the surface of PS@Au led to the presence of a plasmon resonance absorption peak. Meanwhile, MNPAu grew uniformly on the surface, leading to a resonance absorption peak of photonic crystals. The interactions of the two peaks result in the formation of the absorption bands.

The distribution of MNPAu on the surface of the micron gold cage affects the absorption peak position of its UV spectrum^32^. For the first round of growth of MNPAu, micron-sized PS@Au@MNPAu exhibited bimodal absorption in the UV–Vis range. When the volume of the K–H solution was 0.5–7.5 ml, PS@Au@MNPAu showed absorption peaks at approximately 356 and 495 nm. When the volume of the K–H solution was increased to 15 ml, the bimodal absorption peaks were at 429 and 619 nm. This was attributed to a stronger interparticle coupling on a denser gold shell with smaller spacing between nanogold particles, that is, as the gold shell of the micron-sized gold cage became denser with a higher volume of the K–H solution, the absorption peaks were red-shifted^33^. It was also observed that when the volume of the K–H solution used was 15 ml, the FWHM of the bimodal absorption peaks increased (Fig. 3g), which indicated that compared with a less dense nanogold shell, a dense nanogold shell exhibited a stronger coupling with the MNPAu particles.

For the second round of MNPAu growth, when the amount of the K–H solution was 7.5 ml, the bimodal absorption peaks of micron-sized PS@Au@MNPAu also shifted from approximately 356 and 495 nm to approximately 429 and 619 nm, respectively. This showed that with the increase in the number of MNPAu particles on the surface of the micron-sized gold cage, the coupling between the resonance absorption peaks of photonic crystals and the plasmon resonance absorption peaks also took place in the relatively less dense nanogold shell structures. However, the coupling did not always take place with the increase in the number of MNPAu. For the third round of growth of MNPAu, the FWHM of the absorption peaks did not increase in the case of 3.0 ml of the K–H solution. This indicated that for coupling to take place, the nanogold shell should meet a certain density requirement. After the third round of MNPAu growth, the resonance of photonic crystals was enhanced, and their coupling with the plasmon resonance was stronger. This ultimately led to an absorption band of 400–900 nm, confirmed by the spectral analysis in Fig. 3a–c, including plasmon anomalies near 370 nm.

Hereafter, we discuss the purified foam-like gold cage material. An improved multi-step purification method was adopted to remove the PS core of the composite. This was aimed at obtaining a structure with a high purity and a low-density nanogold cavity, which was obtained by employing the following methods, ultraviolet and visible spectrophotometry (UV–Vis), energy dispersive X-ray spectroscopy (EDX), and BET. First, a chemical immersion method was employed, which involved the micron-sized gold cage PS@Au@MNPAu to be added multiple times in a tetrahydrofuran (THF) solution. This ensured the removal of the internal PS template. Subsequently, physical purification was performed by heating the material at 400 °C for 24 h. With the removal of the PS core, the nanogold cavity exhibited a more pronounced broadband absorption.

After the removal of the internal PS template, the density of the hollow, foam-like gold cage materials Au@MNPAu was observed to be in the range of 9–223 mg/cm^3^ and could vary in a continuous and controllable manner with the change in the shell thickness. The purity of the low-density micron gold cage gradually increased during the template removal process, and finally attained the composition of 97.2 wt% Au with 2.8 wt% of C. To the best of our knowledge, this is the highest purity that has been obtained for a low-density gold cage^34^. The average density ρ for a pure micron-sized gold cage can be estimated as.

$$\rho = \rho_{0} \frac{d}{{R_{0} }},$$

where ρ_o_ = 19.3 g/cm^3^ is the density of bulk gold, and d is the average shell thickness. When R_0_ = 10.7 μm (Fig S2) and d = 5 nm (Fig S3), the density is ρ = 9 mg/cm^3^, whereas when R_0_ = 2.6 μm (Fig S2) and d = 30 nm (Fig S3), the density is ρ = 223 mg/cm^3^.

The foam-like gold cage materials synthesized through particle-mediated growth simultaneously possessed dense and loose nanogold shell structures, with the interaction between the two shell structures ensuring that the cages have ultra-wideband absorption. On the other hand, measurement results of the specific surface area revealed that the two nanogold shell structures of foam-like gold cage materials led to an ultra-high specific surface area. Micron-sized gold cages had a BET specific surface area and a Langmuir specific surface area 224 and 1584 times as high as those of gold shells synthesized using seed-mediated growth, respectively (Table1). Based on the assumption that the nanogold particles are spherical with smooth surfaces and same sizes, their surface areas can be associated with the average equivalent particle size by the formula.

$${\text{D}}_{{{\text{BET}}}} \, = \,{6}000/(\rho \, \times \,{\text{S}}_{{\text{w}}} ),$$

where D_BET_ is the average diameter of the nanogold particles (in nm), S_w_ represents the specific surface area of the gold cage powder in m^2^/g, and ρ is the theoretical density of gold^35^. Based on the above formula, the calculations indicate that for nanogold particles having a diameter of 10 nm each, the specific surface area is 31.1 m^2^/g, which is much smaller than the surface area of micro-sized gold cages. The low density of the material was also an important factor that accounted for the broadband absorption in the UV–Vis range. This was because the two nanogold substructures of Au@MNPAu allowed the simultaneous presence of pores with various sizes, such as micropores, mesopores, and macropores.

**Table 1:** BET of micron gold cage and PS@Au.

Surface area	Foam-like gold cage materials (m^2^/g)	PS@Au (m^2^/g)
BET surface area	854.03	3.83
Langmuir surface area	8,394.68	5.30
T-plot micropore area	171.36	1.16
T-plot external surface area	682.67	2.67

**Figure 4:**
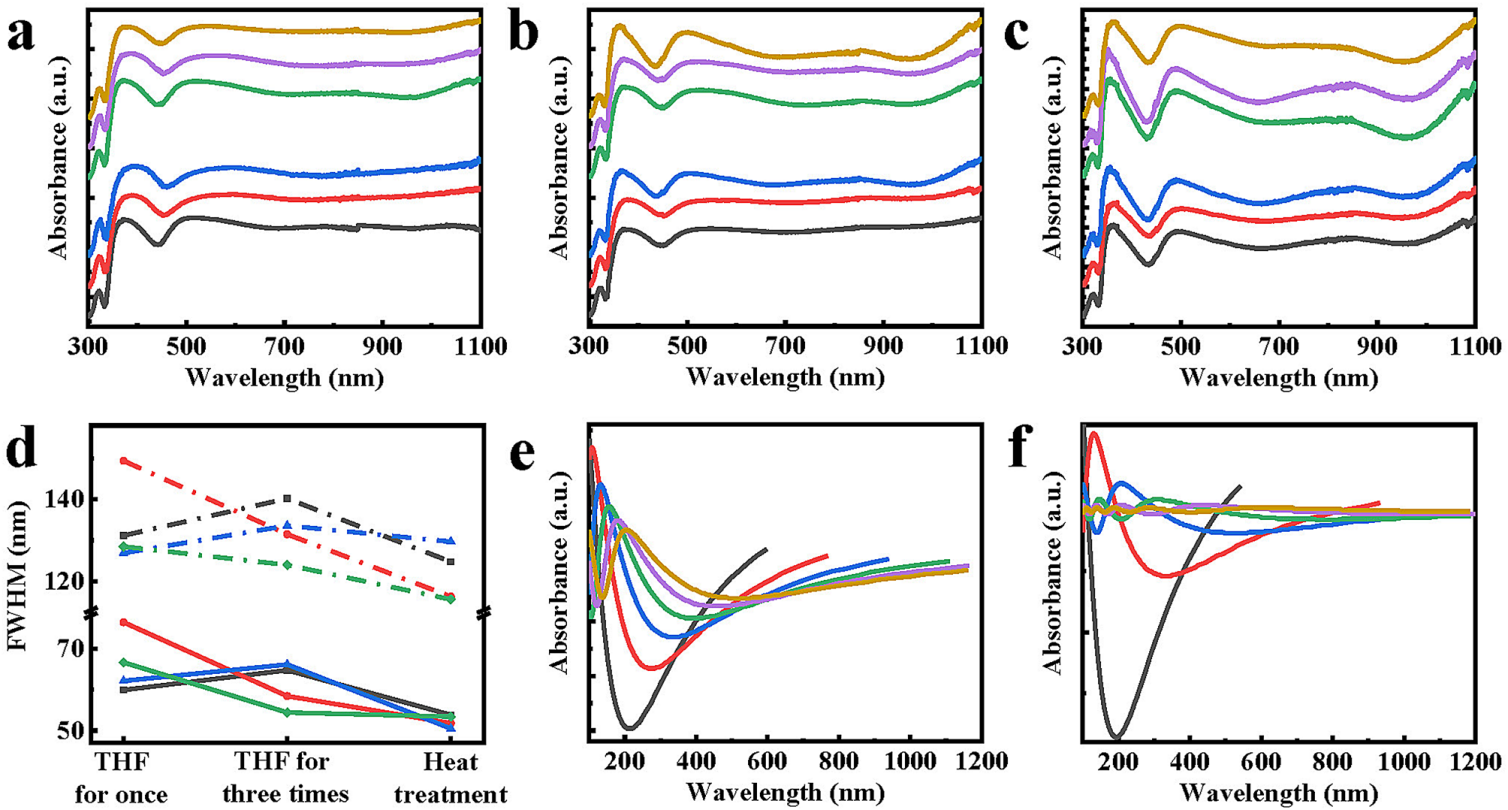
UV–Vis absorption spectra of foam-like gold cage materials during purification. MNPAu was grown once (gray), twice (red), three times (blue) with 7.5 ml of K–H solution, and once (green), twice (purple), three times (yellow) with 15.0 ml of K–H solution, (**a**) immersion in THF, (**b**) three-time immersion in THF, (**c**) heat treatment at 400 °C. (**d**)The FWHM of two absorption peaks of the UV spectrum in the foam-like gold cage varies during purification. MNPAu was grown once (gray) and three times (red) with 7.5 ml of K–H solution, and once (blue) and three times (green) with 15.0 ml K–H solution. (**e**) The mono dispersed golden growth under the conditions d = 6 nm, ƒ_c_ = 0.58, R_i_/R_0_ = 0.53, 0.55, 0.57, 0.59, 0.61, and 0.63 and Λ = 0.7, 0.9, 1.1, 1.3, 1.5, and 1.7, respectively. Here, the residual impurities majorly distribute around the metallic clusters in agglomerates. Therefore, approximately ${\varepsilon }_{i}\simeq 1$ and ${\varepsilon }_{S}=2.5$ are used in our calculations. (**f**) The non mono-dispersed golden growth varies with the diameters d = 5.8, 7.0, 8.2, 9.4, 10.6, and 11.8 at the thermal mass parameter Λ = 0.70. The other values are the same as in (**e**).

The UV–Vis absorption peak in Fig. 4a–c at approximately 370 nm can be attributed to impurities, while the absorption peak at approximately 495 nm could be attributed to plasmon resonance undergoing red shifts as the purification steps proceeded. As shown in Fig. 4c, the nanogold shell of the foam-like gold cage materials exhibited a larger cluster structure after heat treatment, compared with the sample before heat treatment. The cluster structure led to an absorption peak at approximately 850 nm, which can be attributed to photonic crystals. Moreover, when synthesized using 15 ml K–H solution, the sample possessed a thicker shell and a larger cluster structure, and thus, a more pronounced resonance absorption peak of photonic crystals was observed, compared with that of other volumes of the K–H solution. On the other hand, heat treatment led to a decrease in the localized interaction of the nanogold shell, which resulted in three absorption bands that peaked at approximately 370, 495, and 850 nm. These peaks could be attributed to impurities, plasmon resonance, and photonic crystal resonance, respectively.

Furthermore, the FWHM gradually decreased in Fig. 4d, indicating a reduction in the impurity content of foam-like gold cage materials, which was also verified by the gradual increase of Au content in the nanogold cavity structure during the purification process. By considering the thermal deformations of the samples and the residual impurity in the cluster and the agglomerates, the simulations reveal that the anomalous plasmon absorbance is a function of Λ and d with the waveguide effects in accordance with experimental observations qualitatively, as shown in Fig. 4e–f. Notably, the plasma coherence superposition of phase and phase separations near the ultraviolet regime at the structural transitions exists, relying on the sample geometries in sizes and linking to the two-point correlations^27^. The ultraviolet absorption was observed by Thomas et al.^12, 30^, but the peaks can’t be probed since the MNPAu samples exhibited the plasmon correlation effects^18^.

**Figure 5:**
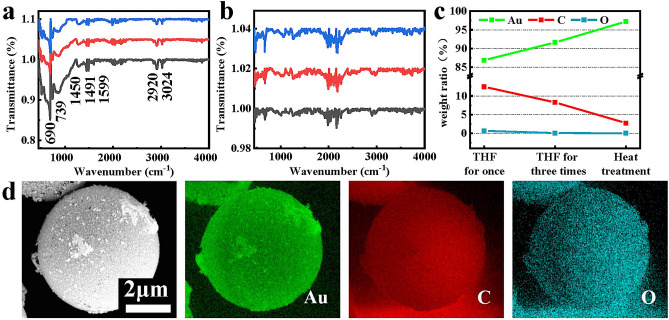
(**a**) Far-infrared absorption spectra of foam-like gold cage materials before THF immersion. (**b**) Far-infrared absorption spectra of foam-like gold cage materials after three-time THF immersion. (**c**) Line charts of the elemental content of Au, O, and C in the purification process of foam-like gold cage materials using the EDX test. (**d**) Elemental distribution diagrams of Au, C, and O after the purification of foam-like gold cage materials.

Far-absorption spectroscopy and elemental content distributions are displayed for the foam-like gold cage materials in Fig. 5a–d. There is an absorption peak at approximately 1600 cm^−1^ in Fig. 5a, which is attributed to the characteristic absorption of the benzene ring skeleton. The absorption at 740–690 cm^−1^ was attributed to the bending vibration of the mono-substituted benzene ring. The obvious absorption peaks at approximately 2800 cm^−1^, in the spectra of Fig. 5a, indicated the presence of symmetric and asymmetric stretching vibrations of C–H in the structure. The absorption peaks at approximately 1450 cm^−1^ and 1490 cm^−1^ could be attributed to the bending vibration of C–H. In Fig. 5b, the absorption peaks of the benzene ring and C–H both disappeared, indicative of complete removal of the PS template from the foam-like gold cage materials after THF immersion. This confirms the change in the elemental Au, C, and O content distribution, as seen in Fig. 5c–d. After the first immersion in THF, most of the PS template in the micron gold cage was removed. The measurements of the zeta potential indicate that the MNPAu structures are stable, as shown in Table 2.

**Table 2:** Zeta potential of MNPAu, PS@Au, and PS@Au@MNPAu.

	zeta potential 1 (mV)	zeta potential 2 (mV)	zeta potential 3 (mV)	Average value (mV)
10 nm MNPAu	− 27.3	− 25.2	− 26.4	− 26.3
PS@Au	− 56.4	− 57.3	− 55.2	− 56.3
PS@Au@MNPAu one time growth MNPAu	− 22.4	− 20.1	− 21.6	− 21.4
PS@Au@MNPAu three-time growth of MNPAu	− 23.4	− 21.4	− 21.8	− 22.2

## Conclusions

Micron-sized core–shell composites with single-layered nanogold shells, referred to as PS@Au in this study, were synthesized using an improved, surface seed-mediated growth process following the Frens method. UV–Vis absorption peaks of PS@Au with incomplete shell structures were first red-shifted and then blue-shifted with an increase in the volume of the K–H solution used in the synthesis. Nanogold core–shell composites PS@Au@MNPAu were synthesized, and managed to overcome the shortcomings of PS@Au, namely a lack of uniform size and nonuniform surface distribution of nanogold on the PS surface. Sample characterization revealed that due to the special structure, PS@Au@MNPAu exhibited notable bimodal absorption in the UV–Vis range. Plasmon resonance absorption peaks of the nanogold shell of PS@Au interacted with the resonance absorption peaks of photonic crystals resulting from MNPAu growth on the surface of PS@Au, ultimately leading to an absorption band ranging from 400–900 nm. The characteristic absorption spectra could be controlled by adjusting the shell geometries in terms of sizes and dielectric environments. Finally, by purifying the PS core with an impurity-specific removal method, a nanogold cavity structure was prepared, which exhibited excellent properties such as high purity, low density, and high specific surface area, resulting in new plasmon anomalous absorption bands that peaked at 370, 495, and 850 nm in the ultraviolet-near infrared range.

In the framework of the Mie-Maxwell–Garnett theories and the Gorkov-Eliashberg (GE) theory for small golden particles, an effective dielectric function of the metallic core–shell has been derived. Numerical simulations for plasmonic absorption have been carried out for describing the tunable absorption phenomena in the UV–Vis range. The results indicate that there exist the LSPRs and the photonic bands crossovers near the percolation threshold. This can be understood as the novel form of the topological transitions with the Gaussian symplectic symmetry, which is consistent with the experimental measurements.

## Experiment

### Growth of PS @ Au @ MNPAu

First, the seed growth method was used. PS@Au core–shell precipitate (1 mL) and deionized water (50 mL) were stirred vigorously for 5 min. PEI (polyethyleneimine) (500 μL) was added to the container and stirred vigorously for 24 h, modifying the PS @ Au microsphere emulsion. The surface was charged, and the remaining solution was centrifuged and washed to obtain a modified PS@Au microsphere emulsion. The excess MNPAu concentrated solution and the modified PS@Au microsphere emulsion were mixed and stirred for 24 h, to ensure that MNPAu adhered to the surface of the PS@Au microsphere by electrostatic action, resulting in a PS@Au microsphere coated with MNPAu. This step was repeated to grow the shells.

### Preparation of foam-like gold cage materials

Based on PS@Au@MNPAu, the PS template was removed by two methods. (a) First, PS@Au@MNPAu (0.1 g) was placed in a test tube filled with the THF solution (10 ml), soaked at room temperature for 24 h. Thereafter, the mixed solution was placed in an ultrasonic cleaner (frequency 40 kHz, duration 3 h), the PS core has been further dissolved in the THF solution under the ultrasonic environment. Finally, the gold cage materials were separated from the upper layer liquid using a centrifuge (rotation speed 3000 rpm, duration 3 min), and the samples were washed six times with deionized water. These steps were repeated three times to ensure that most of the PS are removed. (b) The processed samples were placed in a sintering furnace and subjected to heat treatment in an N_2_ atmosphere. After 3 h, the linear temperature increased to 400 °C and then emained stable for 12 h.

### Sample characterization

The morphologies of the PS @ Au @ MNPAu structures with different nanojunctions were examined using field-emission SEM (GeminiSEM 300, Carl Zeiss). The extinction spectra were measured by a UV–Vis–NIR absorption spectrophotometer (Lambda 35, PerkinElmer). A fourier transform infrared spectrometer (VERTEX 70, Bruker) was used to measure the change in the infrared spectrum of the gold cage material before and after removing the PS template. The specific surface area of Au @ MNPAu was measured using a fully auto surface area analyzer (3Flex, Micromeritics).

### Theory

#### Metallic cage dielectric function

The Mie’s dipolar absorption formula can be extended by solving Poisson Equation^24^. An effective permeability $\stackrel{-}{\varepsilon }$ approaches.

$$\frac{\stackrel{-}{\varepsilon }}{{\varepsilon }_{e}}=\frac{3{\varepsilon }_{i}}{2{\varepsilon }_{m}+{\varepsilon }_{i}}+\left[{\left(\frac{{R}_{0}}{{R}_{i}}\right)}^{3}-1\right]-6\frac{{\varepsilon }_{m}-{\varepsilon }_{i}}{2{\varepsilon }_{m}+{\varepsilon }_{i}}\mathrm{log}\left(\frac{{R}_{0}}{{R}_{i}}\right)$$

where $${\varepsilon }_{e}$$ is the function of the ratio $$\frac{{R}_{0}}{{R}_{i}}$$ of the outside radius $${R}_{0}$$ and the internal radius $${R}_{i}$$, and the dielectric environments $${\varepsilon }_{a}\simeq 1$$ in air or water and the insulator $${\varepsilon }_{i}\simeq 2.5$$ before the purification and $${\varepsilon }_{i}\simeq 1$$ after. The metallic shell permeability $${\varepsilon }_{m}$$ in terms of the Maxwell–Garnett formula, is given as $$\varepsilon_{m} = \varepsilon_{s} \frac{{{\raise0.7ex\hbox{${1 - {\text{f}}_{c} + 3{\text{f}}_{c} \varepsilon_{c} }$} \!\mathord{\left/ {\vphantom {{1 - {\text{f}}_{c} + 3{\text{f}}_{c} \varepsilon_{c} } {\left( {\varepsilon_{c} + 2\varepsilon_{s} } \right)}}}\right.\kern-\nulldelimiterspace} \!\lower0.7ex\hbox{${\left( {\varepsilon_{c} + 2\varepsilon_{s} } \right)}$}}}}{{{\raise0.7ex\hbox{${1 - {\text{f}}_{c} + 3{\text{f}}_{c} \varepsilon_{c} }$} \!\mathord{\left/ {\vphantom {{1 - {\text{f}}_{c} + 3{\text{f}}_{c} \varepsilon_{c} } {\left( {\varepsilon_{c} + 2\varepsilon_{s} } \right)}}}\right.\kern-\nulldelimiterspace} \!\lower0.7ex\hbox{${\left( {\varepsilon_{c} + 2\varepsilon_{s} } \right)}$}}}}$$. Here, $${\varepsilon }_{s}$$ is the permeability of subtract, approximateiy $${\varepsilon }_{s}=2.5$$ as the impurity for simplicity and $${\text{f}}_{c}$$ is the filling factor of the clusters^28^, which characterizes the fractal and the percolation structures near the threshold $${\text{f}}_{c} = 0.5$$, with the permeability $$\varepsilon_{c} = \frac{{1 + \frac{8\pi }{3}{\text{f}}_{c} \chi_{e} }}{{1 - \frac{4\pi }{3}{\text{f}}_{c} \chi_{e} }}$$. The electronic susceptibility $${\chi }_{e}$$ can be linked to the GE susceptibility $${\chi }_{GE}$$ through the relation^29^, $${\chi }_{e}=\frac{{\chi }_{GE}}{1+\frac{4\pi }{3}{\chi }_{GE}}$$. The GE susceptibility^27^ is given by $${\chi }_{GE}=\frac{1}{20\pi }\frac{{e}^{2}}{\Delta d}+\frac{139\Lambda A}{1200{\pi }^{2}{k}_{f}{a}_{B}}$$ with the topological state anomalies $${\chi }_{GE}=\left(\frac{1}{20\pi }\frac{h}{\Delta d}+\frac{139\Lambda hA}{1200{\pi }^{2}{k}_{f}{a}_{B}{e}^{2}}\right)\frac{{e}^{2}}{h}$$ at the structural transitions, where $$e$$ is the charge of an electron, $${a}_{B}$$ is the Bohr radius, $${k}_{f}$$ is the Fermi wave vector of gold, and $$\Lambda =\frac{{m}^{*}}{m}$$ is the thermal effective mass of electron in plasmonically heated nanoparticles^30^. The average level spacing $$\Delta =\frac{12\pi {a}_{B}{e}^{2}}{g\Lambda {k}_{f}{d}^{3}}$$ is the inverse of the density of states per spin near Fermi level and $$g=1+\frac{3{\pi }^{2}}{2{k}_{f}d}+\frac{2}{{\left({k}_{f}d\right)}^{2}}$$ is the modified factor^25, 26^. In such situations, the spin-orbital couplings and the TE/TM mode hybridization link to the frequency-dependent two-point correlation function A in the Gaussian symplectic ensemble^36, 37^.

#### Plasmon anomalous absorbance

For the non-magnetic dielectric cavity in the literatures^28^, the reduced absorbance takes the expression.

$${\upsigma }_{abs}=\frac{k}{{k}_{0}}\frac{\mathrm{\rm I}m\stackrel{-}{\varepsilon }}{{\left|\stackrel{-}{\varepsilon }+2\right|}^{2}}$$

where ${k}_{0}$ is a topological number of wave. It can be determined by the geometric sizes such as the gold particles and the clusters. In the thin shell limit, the term $\frac{\mathrm{\rm I}m\stackrel{-}{\varepsilon }}{{\left|\stackrel{-}{\varepsilon }+2\right|}^{2}}$ varies slowly, the absorbance could exhibit new plasmonic anomalies due to strong plasmon correlations in the submicron scales. In the thick shell limit, the absorbance could display the photonic bands near the percolation threshold with the fractal structure in the ultraviolet-near infrared range. By adjusting the sample geometries in sizes, the LSPRs and the photonic bands could coexist in the crossover regime at the structural transitions.

## Supplementary information

Supplementary file1
