# Supplementary material for: High-purity foam-like micron-sized gold cage material with tunable plasmon properties
Source: Sci Rep. 2020 Oct 6;10:16555. doi: 10.1038/s41598-020-72831-9 (PMC7538574; doi:10.1038/s41598-020-72831-9)
Supplement: Supplementary file 1 — Supplementary file1 [file 41598_2020_72831_MOESM1_ESM.pdf]

Supporting Information

High-purity foam-like micron-sized gold cage material with tunable plasmon properties

Shuo Dong[1], Lin Yi[1,*], Lexiao Cheng[1], Shijian Li[1], Weiming Yang[2], Zhebin Wang[2], and Shaoen Jiang[2]

[1]School of Physics, Huazhong University of Science and Technology, Wuhan, Hubei, 430074, China

[2]Laser Fusion Research Center, China Academy of Engineering Physics, Mianyang, Sichuan, 621900, China

*yilin@hust.edu.cn

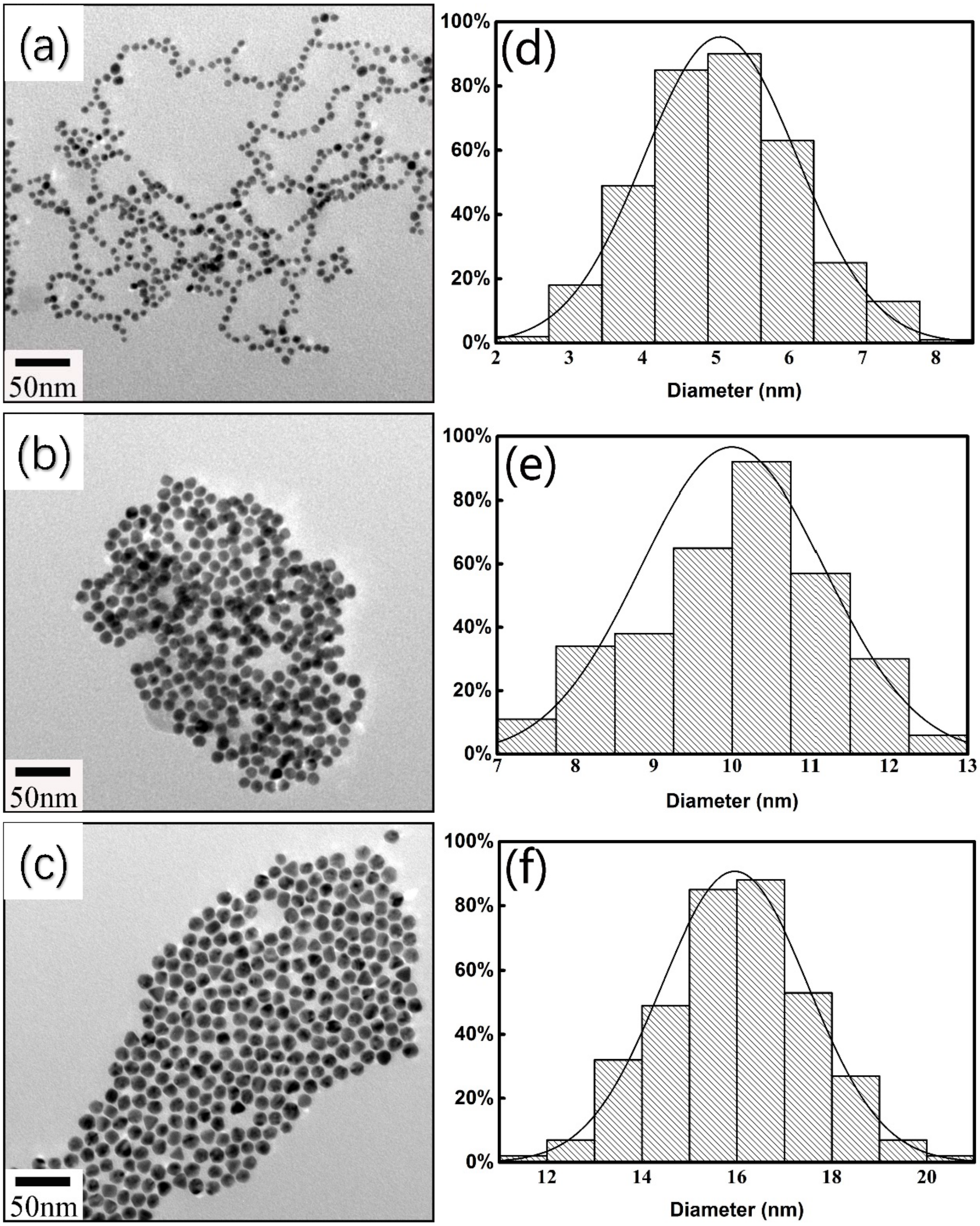

Fig S1. Monodispersed nanoparticles of gold (MNPAu) with different particle sizes (a)5nm, (b)10nm, (c)16nm; (d-f) their particle size distributions.

Table S1. Amount of medicament for MNPAu with different particle sizes

Particle size	$HAuCl_4$	Sodium citrate	K_2CO_3	Tannic acid	Ultrapure water
5nm	1ml	4ml	200μl	700μl	15.10ml
10nm	1ml	4ml	25μl	100μl	15.88ml
16nm	1ml	4ml	2.5μl	20μl	15.98ml

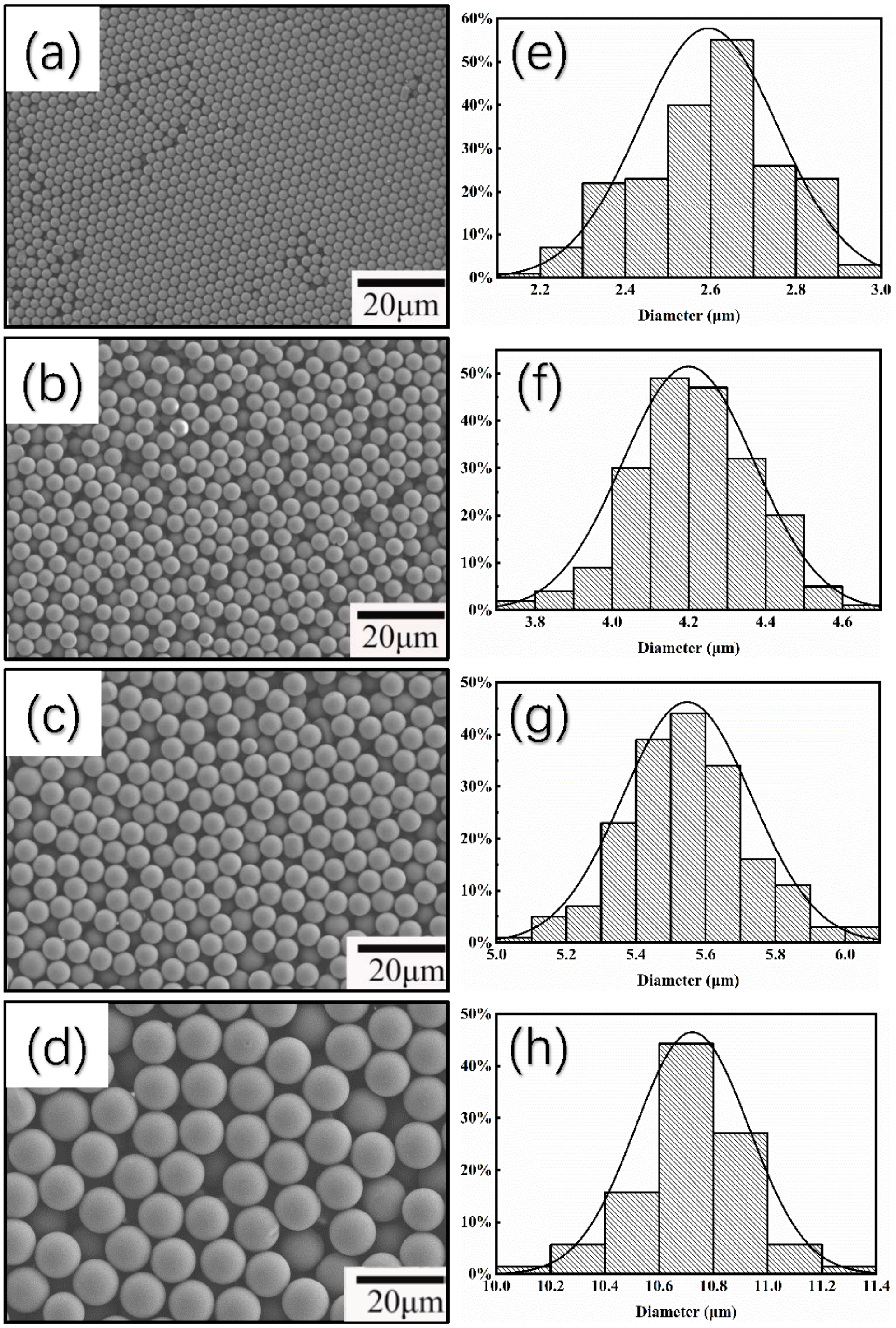

Fig S2. Polystyrene microspheres (PS) with different particle sizes (a)2.6μm, (b)4.2μm, (c)5.5μm, (d)10.7μm; (e-h) their particle size distributions.

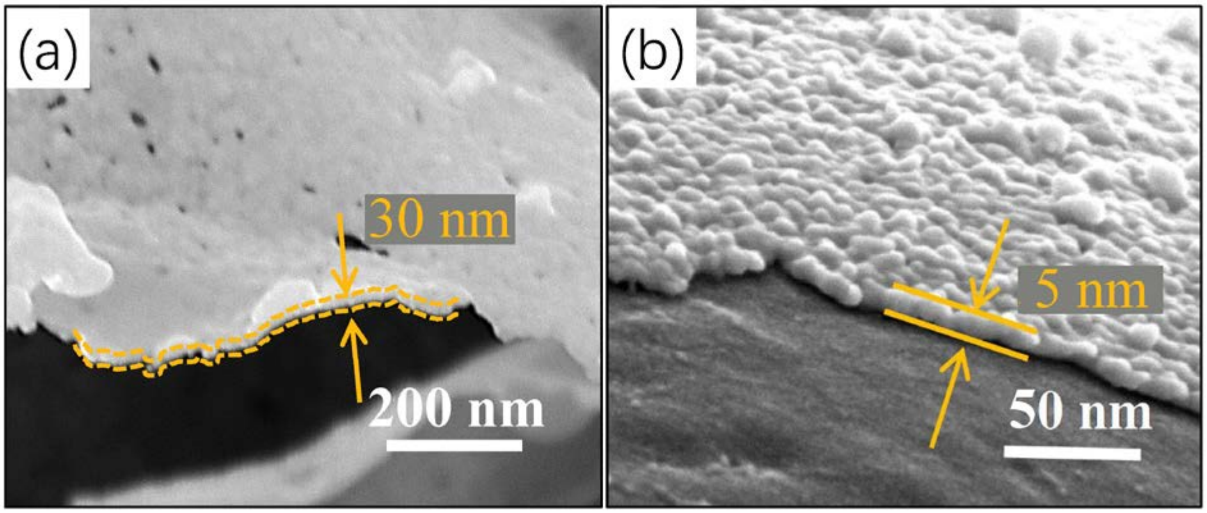

Fig S3. Shell thickness of micron-sized gold cages; (a) the thickness of the shell layer is 30nm with 15ml K-H solution, (b) the thickness of the shell layer is 5nm with 3ml K-H solution.

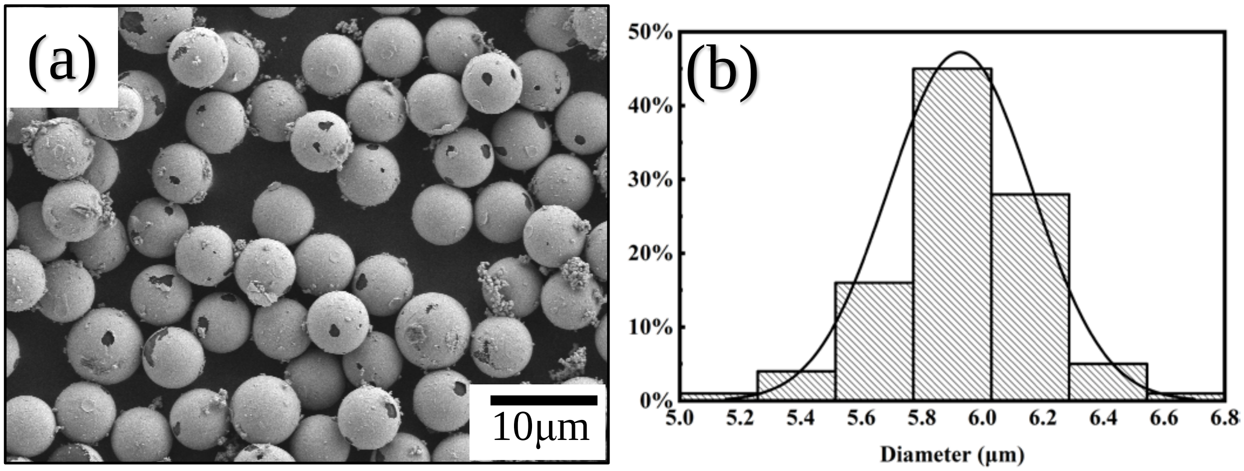

Fig S4. (a) SEM image of micron-sized gold cages; (b) their size distributions.

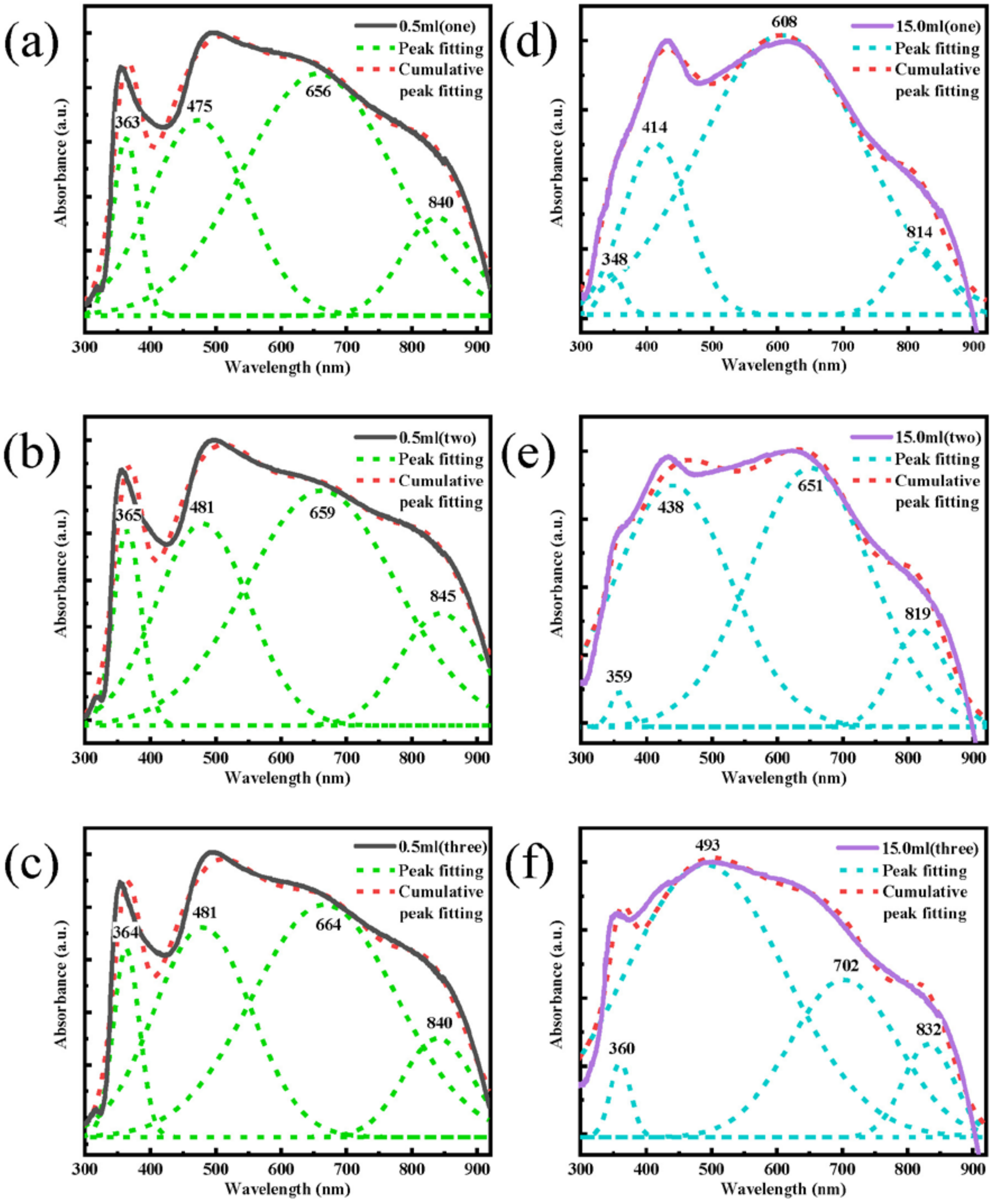

Fig S5. Deconvolution of UV-Vis absorption spectra with PS@Au@MNPAu after one round, two rounds, and three rounds of growth of MNPAu. The amount of K-H solution used in the micron gold cage is 0.5 (a-c) and 15.0 ml (d-f).

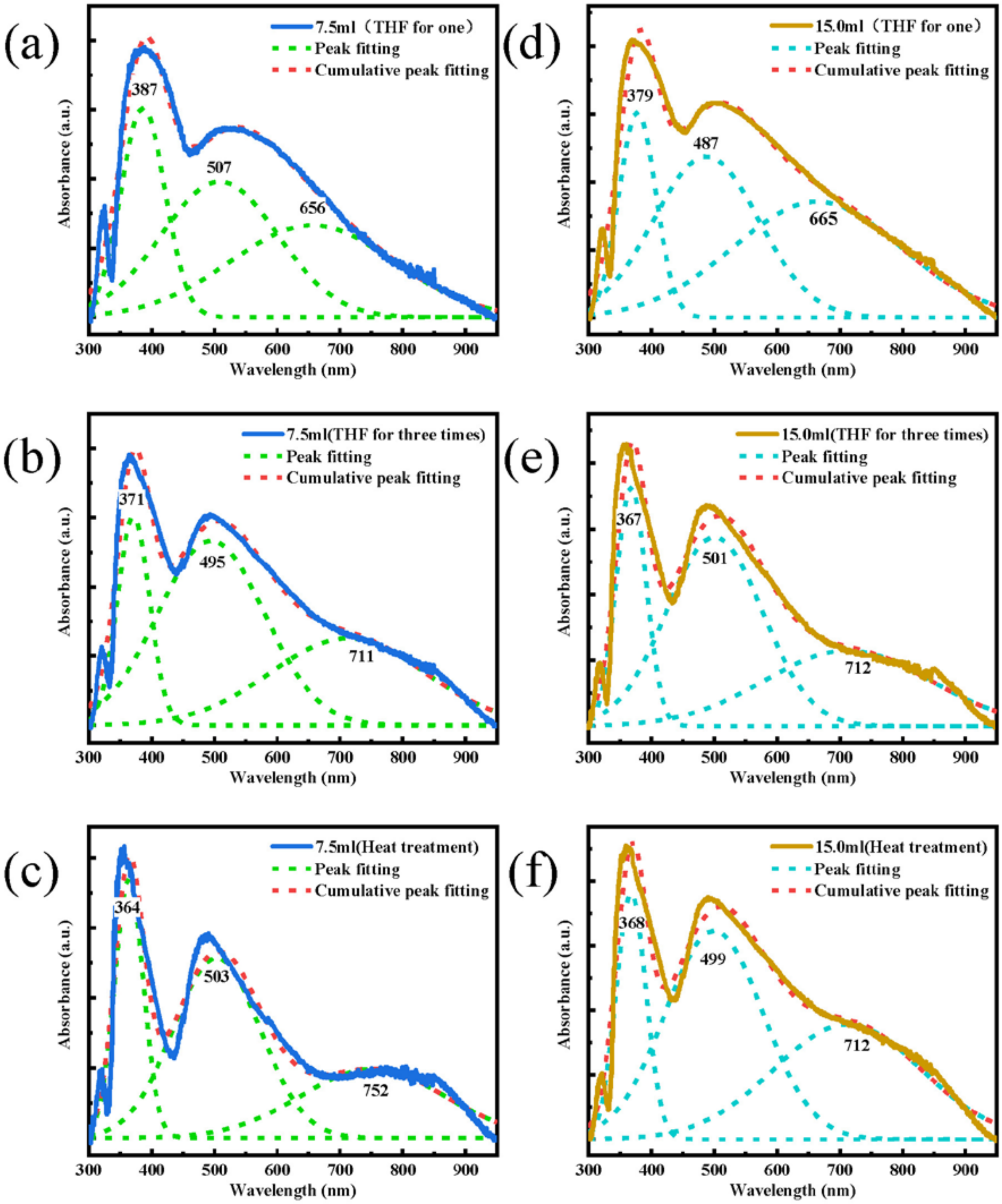

Fig S6. UV spectral baseline leveling and deconvolution of foam-like gold cage materials during purification. MNPAu was grown three times with 7.5 (a-c) and 15.0 ml (d-f) K-H solution.

Theoretical Methods

Theoretical method: Effective Medium Model

In this work, we would like to link the classical Mie-Maxwell-Garnett field theories to the Gorkov-Eliashberg (GE) quantum theory so that to approach the multiscale localized plasmonic spectroscopy. To the end, the model is illuminated by uniform induced electromagnetic fields, with a time-harmonic variation. The Mie's dipolar absorption formula can be extended by solving Poisson equation, with the usual boundary conditions in the literatures. All terms in the multipolar expansion of the potential vanish, except for the dipolar one. After the radial average to electrostatic fields, the permeability $\bar{\epsilon}$ of the cavity reads

$$\frac{\bar{\epsilon}}{\epsilon_{\rm e}} = \frac{3\epsilon_{\rm i}}{2\epsilon_{\rm m}+\epsilon_{\rm i}} + \left[\left(\frac{R_0}{R_{\rm i}}\right)^3 - 1\right] - 6\frac{\epsilon_{\rm m}-\epsilon_{\rm i}}{2\epsilon_{\rm m}+\epsilon_{\rm i}}\log(\frac{R_0}{R_{\rm i}}). \tag{1}$$

and

$$\epsilon_{\rm e} = \frac{3\epsilon_{\rm a}\epsilon_{\rm m}}{2\epsilon_{\rm a}+\epsilon_{\rm m}}\left\{\frac{3\epsilon_{\rm m}}{2\epsilon_{\rm m}+\epsilon_{\rm i}}\frac{2\epsilon_{\rm a}+\epsilon_{\rm i}}{2\epsilon_{\rm a}+\epsilon_{\rm m}} + \left[\left(\frac{R_0}{R_{\rm i}}\right)^3 - 1\right]\right\}^{-1}. \tag{2}$$

Here, the effective permeability $\bar{\epsilon}$ is the function of $\frac{R_0}{R_{\rm i}}$, relying on $\epsilon_{\rm a}$ in air or water, $\epsilon_{\rm i} = 2.5$ for pure PS. The metallic shell permeability $\epsilon_{\rm m}$ takes the Maxwell-Garnett expression

$$\epsilon_{\rm m} \;=\; \epsilon_{\rm s}\frac{1-f_{\rm c}+3f_{\rm c}\epsilon_{\rm c}/(\epsilon_{\rm c}+2\epsilon_{\rm s})}{1-f_{\rm c}+3f_{\rm c}\epsilon_{\rm s}/(\epsilon_{\rm c}+2\epsilon_{\rm s})}. \tag{3}$$

Here, $\epsilon_{\rm s}$ the permeability of subtract, giving the limit of $\epsilon_s = 1$. The effects of the impurities and the structures such as the fractal and the percolation are characterized by the insulator ϵ_i and the filling factor f_c. By considering further the effects of the depolarizing field and the Fano effects, the Tanner-Sievers-Buhrman approximations are used for calculating the cluster permeability

$$\epsilon_{\rm c} = \frac{1+\frac{8\pi}{3}f_c\chi_{\rm e}}{1-\frac{4\pi}{3}f_c\chi_{\rm e}}. \tag{4}$$

Here the electronic susceptibility $\chi_{\rm e}$ links to the GE susceptibility $\chi_{\rm GE}$ through the Strassler-Rice-Wyder relation, namely $\chi_{\rm e} = \frac{\chi_{\rm GE}}{1+\frac{4\pi}{3}\chi_{\rm GE}}$. The GE susceptibility can be revised [4], taking

$$\chi_{\rm GE} = \frac{1}{20\pi}\frac{e^2}{\Delta d} + \frac{139\Lambda A}{1200\pi^2 k_{\rm f} a_{\rm B}} = \chi_0\frac{e^2}{h}. \tag{5}$$

where e is the charge of electron and $\Lambda = \frac{m^*}{m}$ is the thermal effective mass parameter of electron. The average level spacing $\Delta = \frac{12\pi a_{\mathrm{B}} e^2}{g\Lambda k_{\mathrm{f}} d^3}$ is the inverse of the density of states per spin near Fermi level and $g = 1 + \frac{3\pi^2}{2k_{\mathrm{f}}d} + \frac{2}{(k_{\mathrm{f}}d)^2}$ is the modified DOS factor [5]. For the gold atoms, the spin-orbital couplings are strong in the Dyson's random matrix theories [2,3,6−8]. Hence, the two-point correlations are universal, with the dynamic factor, taking

$$\begin{aligned} A &= 2 - \frac{\sin 2z}{2z} - \left[\frac{\pi}{2} + \mathrm{Si}z\right] \frac{\cos z + z\sin z}{z} \\ &+ i\left\{z - \frac{\sin^2 z}{z} + \left[\frac{\pi}{2} + \mathrm{Si}z\right] \frac{z\cos z - \sin z}{z}\right\}. \end{aligned} \tag{6}$$

Here $z = \frac{2\pi\hbar\omega}{\Delta}$ and $\mathrm{Si}z = \int_0^z \frac{\sin y}{y} dy$ is usual sine integral function. The dynamic factor in Eq. (6) is a function of $z = \frac{2\pi\hbar\omega}{\Delta}$, with the dielectric function. For a lossy and the non-magnetic dielectric, the absorption cross section approaches in the literatures [3], taking

$$\bar{\sigma}_{\mathrm{abs}} = 12\pi k R^3 \mathrm{Im}\bar{\epsilon}\frac{1}{\mid \bar{\epsilon} + 2 \mid^2} = \sigma_0 k/k_0 \mathrm{Im}\bar{\epsilon}\frac{1}{\mid \bar{\epsilon} + 2 \mid^2},\ \sigma_{\mathrm{abs}} = \frac{\bar{\sigma}_{\mathrm{abs}}}{\sigma_0} = \frac{k}{k_0}\frac{\mathrm{Im}\bar{\epsilon}}{\mid \bar{\epsilon} + 2 \mid^2}. \tag{7}$$

where $k = \frac{2\pi}{\lambda}$ is the number of wave and $\sigma_0 = \frac{4\pi R^3 k_0}{3}$ is an effective section. The dimensionless absorption cross section has the novel plasma 2D Hall state anomalies

$$\sigma_{\mathrm{abs}} = \frac{k}{k_0}\frac{\mathrm{Im}\bar{\epsilon}}{\mid \bar{\epsilon} + 2 \mid^2}. \tag{8}$$

Here, k_0 is a parameter, determined by in the data of the sample to the sample.

---

[1] Mie, G. Beiträge zur Optik Truüber Medien Speziell Kolloidaler Metallösungen. Ann. Phys. **25**, 377-445 (1908).

[2] Gorkov, L. & Eliashberg, L. P. Minute Metallic Particles in Electron-Magnetic Field. Sov. Phys.-JETP **21**, 940-947 (1965).

[3] Tanner, D. B.; Sievers, A. J. & Buhrman, R. A. Far-Infrared Absorption in Small Metallic Particles. Phys. Rev. **B 11**, 1330-1341 (1975).

[4] Strassler, S.; Rice, M. J. & Wyder, P. Comment on Gorkov and Eliashberg's Result for the Polarizability of a Minute Mettallic Particle. Phys. Rev. B **6**, 2575-2577 (1972).

[5] Granqvist. C. G. Far Infrared Absorption in Ultrafine Metallic Particles: Calculation Based on Classical and Quantum Mechanics Theories. Z. Physik **B 30**, 29-46 (1978).

[6] Grangvist, C. G.; Buhrman, R. A.; Wyns, J. & Sievers, A. J. Far Infrared Absorption in Ultrafine Al Particles. Phys. Rev. Lett. **37**, 625-629 (1976).

[7] Dyson, F.J. The Three Fold Way Algebraic Structure of Symmetry Groups and Ensembles in Quantum Mechanics. J. Math. Phys. **3**, 1199-1215 (1962).

[8] Mehta, M. L. Random Matrices (Academic Press, INC. 1991).

Proofs of Eqs. (1) and (2)

We consider that the nanoshell is illuminated by infinitely uniform induced field along the radial direction (E_{ind}), with a time-harmonic variation. The usual Mie's formula corresponding to the dipolar absorption can be easily extended by solving Poisson equation. The general solutions use the familiar multipolar expansions in the three radial ranges, taking

$$\begin{aligned}
\phi_I &= \sum_n a_n R^n P_n(\cos\theta),\ R \leq R_i \\
\phi_{II} &= \sum_n (b_n R^n + \frac{c_n}{R^{n+1}}) P_n(\cos\theta),\ R_i < R \leq R_0 \\
\phi_{III} &= -E_{\text{ind}} R P_1(\cos\theta) \\
&+ \sum_n \frac{d_n P_n(\cos\theta)}{R^{n+1}}, R > R_0,
\end{aligned} \tag{9}$$

where E_{ind} is induced electric field in the radial direction [3]. Because the electrostatic potential and the normal component of the displacement vector are continuous at $R = R_{\text{i}}$ and R_0. In addition, the potential is finite at $R = 0$ and the field is uniform at $R = \infty$. Hence, we get

$$\begin{aligned}
\phi_I &= \phi_{II}, \epsilon_i \frac{\partial \phi_I}{\partial R} = \epsilon_m \frac{\partial \phi_{II}}{\partial R},\ R = R_i \\
\phi_{II} &= \phi_{III}, \epsilon_m \frac{\partial \phi_{II}}{\partial R} = \epsilon_a \frac{\partial \phi_{III}}{\partial R},\ R = R_0.
\end{aligned} \tag{10}$$

It is easy to see that all terms in the multipolar expansion of the potential vanish, except for the dipolar one. Their dipolar coefficients satisfy the following equations

$$\begin{aligned}
a_1 &= b_1 + \frac{c_1}{R_i^3}, \frac{\epsilon_i}{\epsilon_m} a_1 = b_1 - \frac{2c_1}{R_i^3}, \\
b_1 + \frac{c_1}{R_0^3} &= -E_{\text{ind}} + \frac{d_1}{R_0^3} \\
\frac{\epsilon_m}{\epsilon_a}[b_1 - \frac{2c_1}{R_0^3}] &= -E_{\text{ind}} - \frac{2d_1}{R_0^3}.
\end{aligned} \tag{11}$$

Solving Eq. (11), we obtain

$$a_1 = \frac{3\epsilon_m}{2\epsilon_m+\epsilon_i}b_1,\ c_1 = R_i^3\frac{\epsilon_m-\epsilon_i}{2\epsilon_m+\epsilon_i}b_1,$$
$$d_1 = \frac{R_0^3}{3}c_d b_1,\ b_1 = -3c_b E_{\rm ind}, \tag{12}$$

where the coefficients, c_d and c_b, are given by

$$c_d = 1-\frac{\epsilon_m}{\epsilon_a}+(1+\frac{2\epsilon_m}{\epsilon_a})\frac{\epsilon_m-\epsilon_i}{2\epsilon_m+\epsilon_i}(\frac{R_i}{R_0})^3$$
$$c_b = \frac{\epsilon_a}{2\epsilon_a+\epsilon_m}\frac{(R_0/R_i)^3}{(\frac{R_0}{R_i})^3+2\frac{\epsilon_m-\epsilon_i}{2\epsilon_m+\epsilon_i}\frac{\epsilon_a-\epsilon_m}{2\epsilon_a+\epsilon_m}}. \tag{13}$$

By using Eqs. (12) and (13), the solutions of the scalar potentials can be written as

$$\phi_I = -3\frac{3\epsilon_m}{2\epsilon_m+\epsilon_i}c_b\vec{E}_{\rm ind}\cdot\vec{R},\ R\le R_i$$
$$\phi_{II} = -3[1+\frac{\epsilon_m-\epsilon_i}{2\epsilon_m+\epsilon_i}(\frac{R_i}{R})^3]c_b\vec{E}_{\rm ind}\cdot\vec{R},\ R_i<R\le R_0$$
$$\phi_{III} = -[1+(\frac{R_0}{R})^3c_d c_b]\vec{E}_{\rm ind}\cdot\vec{R},\ R>R_0, \tag{14}$$

The electric field ($\vec{E}=-\nabla\phi$) can be obtained readily

$$\vec{E}_I = 3\frac{3\epsilon_m}{2\epsilon_m+\epsilon_i}c_b\vec{E}_{\rm ind},\ R\le R_i$$
$$\vec{E}_{II} = 3[1-2\frac{\epsilon_m-\epsilon_i}{2\epsilon_m+\epsilon_i}(\frac{R_i}{R})^3]c_b\vec{E}_{\rm ind},\ R_i<R\le R_0$$
$$\vec{E}_{III} = [1-2(\frac{R_0}{R})^3c_d c_b]\vec{E}_{\rm ind}, R>R_0. \tag{15}$$

Here, the conventional radial approximations to the dipolar field is used for the shells in the literatures [3]. By means of the relationship between the displacement and electric field, $\vec{D}=\epsilon\vec{E}$, the radial average dielectric function, $\bar{\epsilon}=\frac{\int_0^{R_0}\epsilon(R)R^2dR}{\int_0^{R_0}R^2dR}$, can be obtained readily

$$\bar{\epsilon} = 3\epsilon_i\frac{3\epsilon_m}{2\epsilon_m+\epsilon_i}c_b(\frac{R_i}{R_0})^3+3\epsilon_m[1-(\frac{R_i}{R_0})^3]c_b$$
$$- 18\epsilon_m\frac{\epsilon_m-\epsilon_i}{2\epsilon_m+\epsilon_i}(\frac{R_i}{R_0})^3c_b\log(\frac{R_0}{R_i}), \tag{16}$$

Suppose $\epsilon_{\rm e}=3\epsilon_m c_b(\frac{R_i}{R_0})^3$, Eq. (16) can be simplified into the expressions in Eqs. (1) and (2).
